# Effects of 8 weeks of Xpand® 2X pre workout supplementation on skeletal muscle hypertrophy, lean body mass, and strength in resistance trained males

**DOI:** 10.1186/1550-2783-10-44

**Published:** 2013-10-09

**Authors:** Ryan P Lowery, Jordan M Joy, Joshua E Dudeck, Eduardo Oliveira de Souza, Sean A McCleary, Shawn Wells, Robert Wildman, Jacob M Wilson

**Affiliations:** 1 Department of Health Sciences and Human Performance, The University of Tampa, Tampa, FL, USA; 2 Laboratory of Neuromuscular Adaptations to Strength Training, School of Physical Education and Sport, University of São Paulo, São Paulo, Brazil; 3 Dymatize® Enterprises, LLC. & Dymatize® Nutrition Sport Performance Institute (DNSPI), Dallas, Texas, USA

**Keywords:** Pre-workout, Performance, Hypertrophy, Supplementation, Sports nutrition

## Abstract

**Background:**

Xpand® 2X is a proprietary blend comprised of branched chain amino acids, creatine monohydrate, beta-alanine (CarnoSyn®), quercetin, coenzymated B-vitamins, alanyl-glutamine (Sustamine®), and natural nitrate sources from pomegranate and beet root extracts purported to enhance the neuromuscular adaptations of resistance training. However to date, no long-term studies have been conducted with this supplement. The purpose of this study was to investigate the effects of a multi-ingredient performance supplement (MIPS) on skeletal muscle hypertrophy, lean body mass and lower body strength in resistance-trained males.

**Methods:**

Twenty resistance-trained males (21.3 ± 1.9 years) were randomly assigned to consume a MIPS or a placebo of equal weight and volume (food-grade orange flavors and sweeteners) in a double-blind manner, 30 minutes prior to exercise. All subjects participated in an 8-week, 3-day per week, periodized, resistance-training program that was split-focused on multi-joint movements such as leg press, bench press, and bent-over rows. Ultrasonography measured muscle thickness of the quadriceps, dual-energy X-ray absorptiometry (DEXA) determined lean body mass, and strength of the bench press and leg press were determined at weeks 0, 4, and 8 of the study. Data were analyzed with a 2 × 3 repeated measures ANOVA with LSD post hoc tests utilized to locate differences.

**Results:**

There was a significant group-by-time interaction in which the MIPS supplementation resulted in a significant (p < 0.01) increase in strength of the bench press (18.4% vs. 9.6%) compared with placebo after 4 and 8 weeks of training. There were no significant group by time interactions between MIPS supplementation nor the placebo in leg press strength (p = .08). MIPS supplementation also resulted in a significant increase in lean body mass (7.8% vs. 3.6%) and quadriceps muscle thickness (11.8% vs. 4.5%) compared with placebo (group*time, p <0.01).

**Conclusions:**

These results suggest that this MIPS can positively augment adaptations in strength, and skeletal muscle hypertrophy in resistance-trained men.

## Background

Sports nutrition scientists have attempted to augment training induced adaptations through a number of supplementation protocols, which generally attempt to enhance and/or speed skeletal muscle development. Research-supported interventions include the provision of the branched chain amino acids (BCAAs), creatine monohydrate, and several other amino acids and their byproducts [1]. Additionally, it has been postulated that a critical period for supplementation is prior (e.g., 30–60 minutes before) to the workout [2]. However, little research have been done on the effects of multi-ingredient pre-workout supplementation on long-term changes in skeletal muscle hypertrophy, lean body mass, and performance.

Multi-ingredient performance supplements (MIPS) may be comprised of ingredients such as BCAAs, creatine, beta-alanine, agmatine sulfate, taurine, creatinol-o-phosphate, and a combination of nitrate-containing compounds purported to enhance both acute performance and long-term neuromuscular adaptations [3-5]. Each of these ingredients has been studied individually for their positive effects on human performance adaptations. For example, pure creatine monohydrate, beta-alanine, and creatinol-o-phosphate have each been shown to enhance muscle adaptations [6,7]. In addition to supporting skeletal muscle hypertrophy, a vast array of nootropics, which have previously been demonstrated to augment preworkout performance including the combinations of glucuronolactone, tyrosine, caffeine, and *rhodiola rosea* are found within the MIPS [8-10]. Lastly, the MIPS contains agmatine sulfate, pomegranate and beet root extracts, which has been shown to enhance skeletal muscle blood flow [11]. However, information is lacking as to how resistance-training individuals will respond to the combination of these ingredients.

Therefore, the primary purpose of this study was to investigate the effects of 8 weeks of a MIPS in resistance-trained individuals during a periodized resistance training program on skeletal muscle hypertrophy, lean body mass, and strength relative to a placebo matched control.

## Methods

### Overview

Our study consisted of a randomized, double-blind, diet-controlled design, wherein a pre-workout supplemented and a placebo supplemented group were assigned to an 8-week periodized resistance training protocol. Muscle thickness, lean body mass, and strength were examined collectively at the end of weeks 0, 4, and 8 to assess the effects of the MIPS.

### Subjects

Twenty-four resistance-trained males aged 21.3 ± 1.9 years with a respective average leg press and bench press of 3.40 ± 0.2 and 1.38 ± 0.9 times their bodyweight and a minimum of 1 year of resistance training experience were recruited for the study. Subjects could not participate if they were currently taking any medications including anti-inflammatory agents, any performance enhancing supplements, or if they smoked. Specifically, subjects could not have taken nutritional supplements for at least three months prior to data collection. Inclusion criteria included that subjects had previously been performing a minimum of 3 days per week resistance training. Each participant signed an informed consent approved by the University of Tampa Institutional Review Board before participating in the study.

### Resistance training protocol

Our resistance training protocol was a modified combination from Kraemer et al. [12] and Montiero et al. [13]. These researchers found that a non-linear resistance-training program yielded greater results than a traditional or non-periodized program in athletes. The program was designed to train all major muscle groups using mostly compound movements for the upper and lower body (Table 1). The programmed, non-linear training split was divided into hypertrophy days consisting of 8–12 repetition maximum (RM) loads for 3 sets, with 60–120 seconds rest and strength days consisting of 2 to 5 RM loads for 3 sets for all exercises except the leg press and bench press which received 5 total sets. Subjects resistance-trained on Monday, Wednesday, and Friday of each week. Monday and Wednesday consisted of hypertrophy style training while Friday was dedicated to strength. Weights were progressively increased by 2–5% when the prescribed repetitions could be completed. All training sessions were closely monitored to ensure effort and intensity were maximal each training session. A research assistant worked individually with each subject to make sure full range of motion as well as rest intervals and repetition schemes were followed.

**Table 1:** Resistance training protocol

**Monday**	**Wednesday**	**Friday**
*hyper - leg&chest*	*hyper - back&delts*	*strength*
Leg Press	Pullups	Leg Press
Leg Curl	90° Bent Rows	Bench Press
Leg Extension	Shrugs	Leg Extension
Hyperextension	Shoulder Press	Close Grip Bench Press
Bench Press	Lateral Raise	
Dumbbell incline Bench Press	Reverse Laterals	
Close Grip Bench Press	Bicep curls	
Tricep cable extension		
Rep Scheme: 6 - 15	Rep Scheme: 6 - 15	Rep Scheme: 1 - 5

### Strength, lean body mass, and muscle mass testing

Strength was assessed via 1-RM testing of the leg press and bench press. Lean Body Mass was determined on a Lunar Prodigy dual energy x-ray absorptiometry (DEXA) apparatus (software version, enCORE 2008, Madison, Wisconsin, U.S.A.). Skeletal muscle hypertrophy was determined via changes in quadriceps ultrasonography (determined by taking the combined muscle thickness of the *vastus lateralis* (VL) and *vastus intermedius* (VI) muscles. The reliability of measurements of muscle thickness by the same investigator was 0.98. These tests were performed at the end of weeks 0,4, and 8 in replacement of the heavy day in order to ensure full recovery (Friday).

### Supplementation and diet control

Prior to the study, subjects were randomly assigned to consume Xpand® 2X (MIPS) or an isocaloric placebo of equal weight and volume (food-grade orange flavors and sweeteners) in a double-blind manner 30 minutes prior to exercise. On the non-training days, subjects were instructed to consume three equal servings spread evenly throughout the day with breakfast, lunch, and dinner. Two weeks prior to as well as throughout the study, subjects were placed on a diet consisting of 25% protein, 50% carbohydrates, and 25% fat by a registered dietitian who specializes in sport nutrition. Post study, analysis of the subjects recorded diets revealed diets consisted of 23% protein, 46% carbohydrates, and 31% fat, with no differences between groups. Subjects met as a group with the dietitian and were given individual meal plans at the beginning of the study as well as instructed not to consume any alcohol as it can dehydrate and impair the subjects’ results. Upon completion of the study, all subjects’ diets were analyzed to ensure compliance. Since the dietician worked weekly with the subjects, no subject’s data had to be removed due to non-compliance. No adverse side effects were found in either group.

### Statistical analysis

Repeated measures analysis of variance was performed to assess group, time, and group by time interactions. If any main effects were found, an LSD post hoc was employed to locate differences. An alpha priori of p < 0.05 was established. Statistica (StatSoft®, Tulsa, OK, USA) was used for all statistical analysis.

## Results

**Figure 1:**
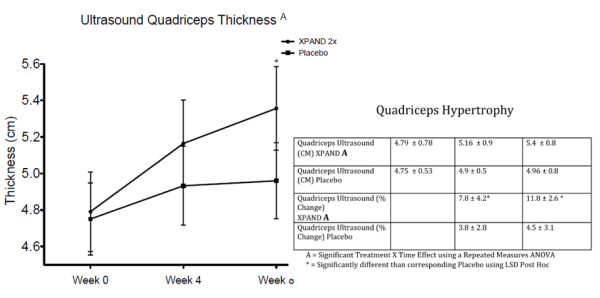
Percent change ultrasound muscle hypertrophy.

**Figure 2:**
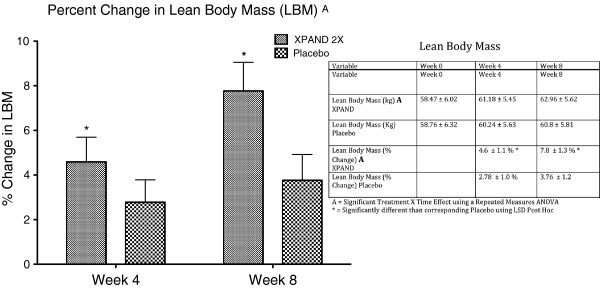
Percent change in lean body mass.

**Figure 3:**
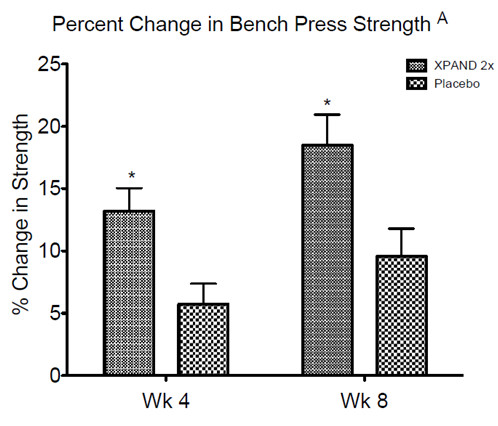
Percent change in bench press strength.

MIPS supplementation contributed to a significant increase in quadriceps muscle thickness compared with placebo (group-by-time, p < 0.01). After 8 weeks of training, the MIPS contributed to quadriceps thickness increases of 11.8%, which was significantly greater than the increases resulting from the training alone in the placebo group of 4.5% (p < 0.01) (Figure 1). In addition, MIPS supplementation contributed to a significant increase in lean body mass (LBM) compared with placebo (group-by-time, p < 0.01). After 8 weeks of training, MIPS supplementation significantly contributed to a LBM increase of 7.8%, which was significantly greater than the increases resulting from the placebo group of 3.6% (p < 0.01) (Figure 2). The MIPS significantly contributed to an increase in bench press strength compared with placebo (group-by-time, p < 0.05). After 8 weeks of training, the MIPS contributed to bench press strength increases of 18.4%, which was significantly greater than the increases resulting from the training alone in the placebo group of 9.6% (p < 0.01) (Figure 3) in which the MIPS group increased 41.4%, while the placebo group increased 34.2%. Lastly, there was a trend toward an increase in leg press strength (group-by-time, p = .08).

## Discussion

The primary purpose of this study was to investigate the effects of 8 weeks of a MIPS in resistance-trained individuals during a periodized resistance training program on skeletal muscle hypertrophy, lean body mass, and strength relative to a placebo matched control. The primary findings of this research were in congruence with our hypotheses that Xpand® 2X supplementation can improve adaptations in skeletal muscle hypertrophy, LBM, and strength.

### Skeletal muscle hypertrophy and lean body mass

This MIPS contains a proprietary blend of ingredients reported previously to augment the accretion of skeletal muscle. For example, creatine monohydrate has been reported as the most effective ergogenic aid currently available regarding LBM and high-intensity exercise capacity, particularly in untrained individuals [14]. Creatine and its various forms have been thoroughly researched, yet to date no study has shown any form of creatine to be superior to creatine monohydrate, which was used in this study (23, 33). Supplementation with creatine can increase total resistance training volume via ATP re-synthesis [14], and total training volume has been closely linked with skeletal muscle accretion. Additionally, creatine supplementation has been demonstrated to increase the activation of satellite cells and myonuclei in muscle following chronic resistance training [15]. Moreover it is conceivable that the osmotic pressure created by creatine that increases the hydration status of cells, resulting in potentially hypertrophic effects [16]. This mechanism of action is what causes creatine to increase strength, but can benefit almost every body system, including the brain, bones, muscles, and liver [17]. Lastly, long term studies have observed that those supplementing with creatine experience 200% increases in LBM compared to placebo [14].

Branched chain amino acids have previously been shown as efficacious in the accretion of skeletal muscle mass [2]. One BCAA of particular interest is leucine, which has been shown to increase muscle protein synthesis (MPS) without the presence of the other essential amino acids [18] Additionally, Karlsson et al. [19] found that supplementation with BCAAs during resistance exercise results in greater phosphorylation of ribosomal S6 kinase, a rate limiting enzyme in the signaling network responsible for regulation of protein synthesis in skeletal muscles. Moreover, BCAAs seem to decrease soreness after eccentric exercise [20] and, they prevent declines in both testosterone and power following an overreaching cycle [21].

Beta-Alanine supplementation has consistently been demonstrated to augment muscle carnosine concentrations in humans [22-25]. Harris et al. [22] concluded that carnosine plays an essential role as an intracellular buffer within the skeletal muscle of humans. More importantly, beta-alanine supplementation has been shown to enhance physical performance during high intensity exercise bouts while also delaying the onset of neuromuscular fatigue [26].

Agmatine is a derivative of the amino acid arginine. Agmatine has been studied for its impact on nitric oxide, wellbeing, and hormone status [27,28]. Agmatine has been noted to support nitric oxide (NO) production via stimulation of endothelial nitric oxide synthase (eNOS). [29-31]. This process is essential for the proper functioning of the polyamine biosynthetic pathways to occur [29-31]. The body’s organs require polyamines for their growth, renewal, and metabolism. Polyamines also have a profound stabilizing effect on a cell DNA and are essential to the healthy function of the nervous system [29,30]. Therefore, these pathways, although not fully elucidated, play an important role in normal cell homeostasis.

Lastly, Creatinol-O-Phosphate (COP) is known primarily for its abilities as an intracellular buffer. Creatinol-O-Phosphate has been shown to assist in stabilizing intracellular and extracellular pH levels, ultimately prolonging anaerobic glycolysis in the presence of lactic acid [7]. Creatinol-O-Phosphate has also been shown to activate satellite cells in skeletal muscle, theoretically increasing their capacity for muscle growth [7]. To date this is the first study that we are aware of to analyze this specific combination of ingredients.

### Strength

Strength is one of the most critical attributes underlying success in sport [32]. The collective results of the present study suggest that bench press strength following Xpand® 2X supplementation are optimized within the context of a periodized training split. Most of the individual ingredients found within the pre-workout matrix have been demonstrated to individually enhance a variety of strength measures. For example, creatine monohydrate has repeatedly been shown to augment strength [33]. In addition to creatine, this MIPS contains a number of nootropics including glucuronolactone, caffeine, and taurine, which can possibly decrease perceived exertion and augment intra-workout performance. For example, caffeine has been demonstrated to enhance strength acutely through an adenosine receptor antagonist mediated mechanism and subsequent central nervous system stimulation [34].

Research suggests that taurine might increase the mechanical threshold for skeletal muscle contraction, promote intracellular membrane stabilization, and increase membrane polarization [22]. Taurine has also been noted for its performance enhancing, antioxidant, and nerve conduction effects [22,35]. Moreover the combination of caffeine and taurine has previously been shown to augment performance to a greater degree than caffeine alone [36].

## Conclusions

The collective results of our research indicate that Xpand® 2X (MIPS) supplementation results in significant increases in bench press strength, hypertrophy, and lean body mass in resistance-trained men. Therefore, coaches, trainers, and athletes may increase the benefits of their workouts by supplementing with MIPS prior to exercise. Future research should investigate if supplementation with MIPS would result in better adaptations than supplementation with each of the ingredients alone.

## Competing interests

RPL, JMJ, JED, EOS, SAM, and JMW declare that they have no competing interests. SW and RW are employed by Dymatize®.

## Authors’ contributions

RPL, JMJ, JED, SAM, and JMW contributed in concept and design, data acquisition, analysis of data, and manuscript preparation. EOS was involved in concept and design, analysis of data, and manuscript preparation. SW and RW were involved in concept and design and manuscript preparation. All authors read and approved the final manuscript.
